# A systematic review and meta-analysis and mendelian randomization analysis of serum phosphorus, albumin, and CRP as risk factors for death in hemodialysis patients

**DOI:** 10.5937/jomb0-53079

**Published:** 2026-03-17

**Authors:** Xiaofen Ma, Huan Ye

**Affiliations:** 1 Zhejiang Hospital, Blood Purification Center, Hangzhou City, Zhejiang Province, China

**Keywords:** renal dialysis, phosphorus, serum albumin, C-reactive protein, mortality, mendelian randomization analysis, bubrežna dijaliza, fosfor, serumski albumin, C-reaktivni protein, mortalitet, Mendelova randomiza-ciona analiza

## Abstract

**Background:**

We aimed to identify the main mortality risk factors in hemodialysis patients using data from relevant cross-sectional literature. We used Mendelian randomization (MR) to assess the causality of those identified risk factors using pertinent Genome-Wide Association Study (GWAS) Single Nucleotide Polymorphism (SNP) data.

**Methods:**

Sixteen publications detailing hemodialysis-related mortality implicated 32 death-related risk factors. Based on heterogeneity testing, we utilized randomand stratified-effects models for meta-analysis. Sensitivity analysis and bias testing were used to evaluate data reliability. Mr analysis identified type-2 diabetes (T2D), serum phosphorus, serum albumin, and age as risk factors, with hematology as the outcome. Inverse-variance weighting (IVW) analysis was used in the main study. The consistency of the IVW analysis results was evaluated simultaneously using four different methods: Mr Egger regression, weighted median estimator (WME), weighted mode, and simple mode. Horizontal pleiotropy was assessed using the Mr Egger regression intercept term; heterogeneity was evaluated using Cochran's Q.

**Results:**

Using randomand stratified-effects models, a meta-analysis of 16 published articles revealed that the following factors were associated with a greater mortality risk in hemodialysis patients: T2D; serum phosphorus, albumin, and CRP; and the Charlson comorbidity index (CCI). The results were deemed reliable based on bias (P=0.1186, I2=99.53%) and sensitivity (T=0.39, df=116, P=0.6953) analyses. IVW indicated a genetic-level positive causal relationship between T2D and hematology (OR=1.2572, 95% CI=1.0375-1.5235; P=0.0195). Genetic-level serum pathology and hematology were positively correlated (OR=2.0269, 95% CI=1.0614-3.8708; P=0.0323). However, age (OR=11.1112, 95% CI=0.83) was a factor. No discernible genetic causal relationship occurred between hematology and serum ferritin (OR=0.6707, 95% CI=0.4612-0.9707; P=0.4612) or albumin (OR=1.2933, 95% CI=0.8931-1.8729; P=0.1733).

**Conclusions:**

Meta-analysis identified the number of dialysis sessions, serum CRP, CCI, T2D, serum phosphorus, and serum albumin as mortality risk factors in hemodialysis patients. The Mr results showed positive causal relationships between T2D incidence and serum phosphorus with hemodialysis risk.

## Abstract (sr)

**Uvod:**

<span lang="SR-LATN-RS" style="font-size:11.0pt; line-height:115%;font-family:&quot;Calibri&quot;,sans-serif;mso-ascii-theme-font:minor-latin; mso-fareast-font-family:Calibri;mso-fareast-theme-font:minor-latin;mso-hansi-theme-font: minor-latin;mso-bidi-font-family:&quot;Times New Roman&quot;;mso-bidi-theme-font:minor-bidi; mso-ansi-language:SR-LATN-RS;mso-fareast-language:EN-US;mso-bidi-language:AR-SA">Cilj je bio da se identifikuju glavni faktori rizika od smrtnosti kod pacijenata na hemodijalizi koristeći podatke iz relevantne poprečne literature. Koristili smo Mendelovu randomizaciju (MR) da bismo procenili uzročnost tih identifikovanih faktora rizika koristeći relevantne podatke o polimorfizmu jednog nukleotida (SNP) iz studije asocijacije celog genoma (GWAS). </span>

**Metode:**

<span lang="SR-LATN-RS" style="font-size:11.0pt; line-height:115%;font-family:&quot;Calibri&quot;,sans-serif;mso-ascii-theme-font:minor-latin; mso-fareast-font-family:Calibri;mso-fareast-theme-font:minor-latin;mso-hansi-theme-font: minor-latin;mso-bidi-font-family:&quot;Times New Roman&quot;;mso-bidi-theme-font:minor-bidi; mso-ansi-language:SR-LATN-RS;mso-fareast-language:EN-US;mso-bidi-language:AR-SA">Šesnaest publikacija koje detaljno opisuju mortalitet povezan sa hemodijalizom implicirale su 32 faktora rizika povezanih sa smrću. Na osnovu testiranja heterogenosti, koristili smo modele sa slučajnim i stratifikovanim efektima za meta-analizu. Analiza osetljivosti i testiranje pristrasnosti korišćeni su za procenu pouzdanosti podataka. Mr analiza je identifikovala dijabetes tipa 2 (T2D), serumski fosfor, serumski albumin i starost kao faktore rizika, sa hematologijom kao ishodom. U glavnoj studiji korišćena je analiza inverzne varijanse (IVW). Konzistentnost rezultata IVW analize procenjena je istovremeno korišćenjem četiri različite metode: MREgerova regresija, ponderisani medijalni estimator (WME), ponderisani mod i jednostavan mod. Horizontalna pleiotropija je procenjena korišćenjem presečnog člana Mr-Egerove regresije; heterogenost je procenjena korišćenjem Kohranovog Q koeficijenta.</span>

**Rezultati:**

<span lang="SR-LATN-RS" style="font-size:11.0pt; line-height:115%;font-family:&quot;Calibri&quot;,sans-serif;mso-ascii-theme-font:minor-latin; mso-fareast-font-family:Calibri;mso-fareast-theme-font:minor-latin;mso-hansi-theme-font: minor-latin;mso-bidi-font-family:&quot;Times New Roman&quot;;mso-bidi-theme-font:minor-bidi; mso-ansi-language:SR-LATN-RS;mso-fareast-language:EN-US;mso-bidi-language:AR-SA">Koristeći modele slučajnih i stratifikovanih efekata, meta-analiza 16 objavljenih članaka otkrila je da su sledeći faktori povezani sa većim rizikom od smrtnosti kod pacijenata na hemodijalizi: dijabetes tipa 2; serumski fosfor, albumin i CRP; i Čarlsonov indeks komorbiditeta (CCI). Rezultati su ocenjeni pouzdanim na osnovu analiza pristrasnosti (P= 0,1186, I2=99,53%) i osetljivosti (T=0,39, df=116, P= 0,6953). IVW je ukazala na pozitivnu uzročnu vezu na genetskom nivou između dijabetesa tipa 2 i hematologije (OR= 1,2572, 95% CI=1,0375-1,5235; P=0,0195). Patologija seruma i hematologija na genetskom nivou bile su pozitivno korelirane (OR=2,0269, 95% CI=1,0614-3,8708; P= 0,0323). Međutim, starost (OR=11,1112, 95% CI=0,83) je bila faktor. Nije utvrđena uočljiva genetska uzročna veza između hematologije i serumskog feritina (OR=0,6707, 95% CI=0,4612-0,9707; P=0,4612) ili albumina (OR= 1,2933, 95% CI=0,8931-1,8729; P=0,1733).</span>

**Zaključak:**

<span lang="SR-LATN-RS" style="font-size:11.0pt; line-height:115%;font-family:&quot;Calibri&quot;,sans-serif;mso-ascii-theme-font:minor-latin; mso-fareast-font-family:Calibri;mso-fareast-theme-font:minor-latin;mso-hansi-theme-font: minor-latin;mso-bidi-font-family:&quot;Times New Roman&quot;;mso-bidi-theme-font:minor-bidi; mso-ansi-language:SR-LATN-RS;mso-fareast-language:EN-US;mso-bidi-language:AR-SA">Meta-analiza je identifikovala broj sesija dijalize, serumski CRP, CCI, dijabetes tipa 2, serumski fosfor i serumski albumin kao faktore rizika od smrtnosti kod pacijenata na hemodijalizi. Rezultati Mr su pokazali pozitivne uzročne veze između incidence dijabetesa tipa 2 i serumskog fosfora sa rizikom od hemodijalize</span>.

## Introduction

Chronic kidney disease affects over 10% of the global population, disproportionately impacting older adults, women, and those with diabetes and hypertension. Patients receiving dialysis for longer than five years now make up 33.7% of the patient China Research Data Service Platform population [1][2]. Despite advances in dialysis technology and patient care, mortality rates among hemodialysis patients remain high [3][4]. Studies have consistently shown that mortality rates in this population are influenced by a range of factors, including patient demographics, comorbidities, and dialysis treatment characteristics [5][6]. For example, older age, female gender, and the presence of certain comorbidities such as cardiovascular disease and diabetes have been identified as risk factors for mortality in hemodialysis patients [7][8].

Additionally, the dose of hemodialysis is an important predictor of patient mortality, with higher doses associated with lower mortality rates [6][7][8]. However, the relationship between dialysis dose and mortality is complex, and the optimal dose of dialysis remains a topic of debate [7][8]. Furthermore, the causes of death in hemodialysis patients are multifaceted, with infections, cardiovascular disease, and withdrawal from dialysis being common contributing factors [6][7][8]. As a result, it is critical to investigate the risk factors linked to mortality in hemodialysis patients, create individualized plans to improve patient survival in nursing and clinical settings and establish a theoretical framework for forecasting patient mortality risk.

This cross-sectional study aimed to determine the primary risk factors linked to mortality in hemodialysis patients by using valuable literature as a data source. Using the GWAS database as the primary source of SNP data, MR analysis was conducted to investigate the causal relationship between mortality-related risk factors in hemodialysis patients and the genetic risk of hemodialysis.

## Materials and methods

### Overall study design

**Figure 1:**
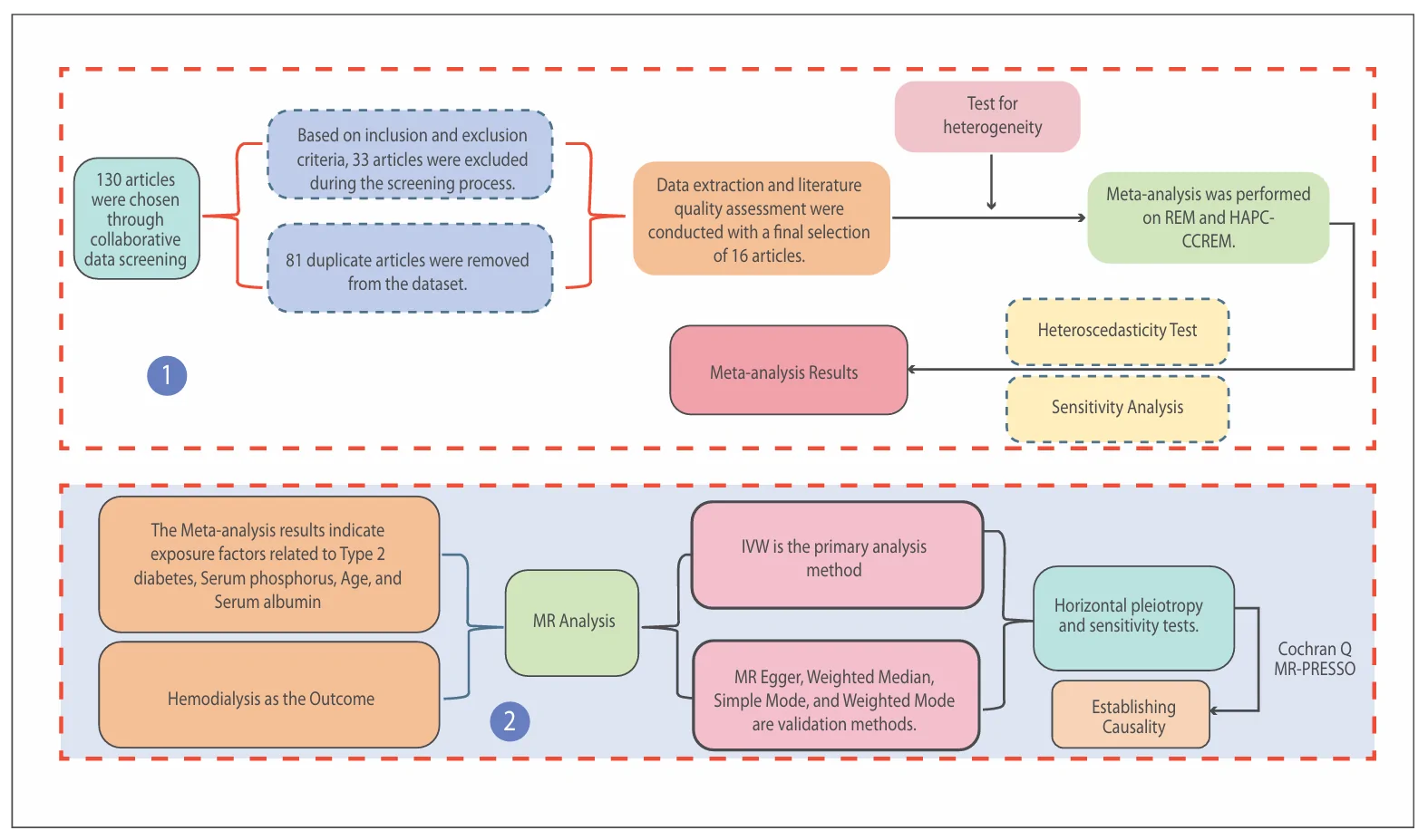
Research Workflow Diagram.

Serum albumin, age, diabetes status, number of dialysis sessions, serum CRP concentrations, serum phosphorus concentrations, and the Charlson comorbidity index (CCI) were consistently identified as mortality risk factors through a systematic analysis of 16 included studies. This study utilized Mendelian randomization (MR) analysis to investigate the genetic causal relationship between relevant variables and hemodialysis. Please refer to Figure 1 for details of the research process.

### Techniques for choosing and examining literature for meta-analysis

### The inclusion, exclusion, and literature search criteria

The main sources of literature used for the literature search were the Cochrane Library, PubMed, Ovid, Web of Science, and EBSCO databases. The search was performed through December 2023. The terms »hemodialysis,« »death,« and »risk factors« were used in English. The search terms »hemodialysis« »hemodialysis death« and »death« or »risk factors« were combined with free words to create the search strategy. The inclusion criteria for this review included complete data from published primary literature, cross-sectional studies with a total number of cases, a reasonable study design, odds ratios (ORs) and 95% confidence intervals (CIs) for outcomes and all of the above. The exclusion criteria for this review were as follows: were written in languages other than English, were basic studies, had incomplete clinical data, lacked indicators related to deaths from hemodialysis, or full-text was not accessible.

### Screening of the literature, data extraction, and quality evaluation

When there was a disagreement between the two individuals screened for inclusion and exclusion criteria, three participants were included in the study. The first author, the year the work was published, the number of patients in the control and observation groups, the interventions used in both groups, the clinical outcomes (total number of deaths or survivors), and quality assessment indicators such as blinding, randomization, and loss to follow-up in the study were among the basic literature details included in the data extraction process. The PEDro scale, a technique for assessing trial quality, was used to gauge the caliber of the included studies. Due to the inclusion criteria and participant external validity, the first question was not included in determining the overall PEDro score. As such, there are 11 questions on the scale overall, but only 10 of them can be scored. The PEDro scale is a useful tool for assessing data in scientific research and is frequently used to evaluate the risk of bias. Studies are categorized as high quality when they receive a PEDro score of 6 to 10, as moderate quality when they receive a score of 4 to 5, and as low quality when they receive a score of 0 to 3.

### Statistical evaluation

Using RevMan 5.3, a meta-analysis of the extracted data was carried out. Odds ratios (ORs) with 95% confidence intervals (CIs) were used to express the statistical effect sizes for counts, and mean differences (MDs) with 95% confidence intervals (CIs) were used to express the statistical effect sizes for continuous data. When I^2^ ≤ 50% suggested homogeneity, a fixed-effects model was utilized for the meta-analysis; when heterogeneity was present, a random-effects model was used. Sensitivity analyses were performed using different effect models and were used to evaluate the stability of the pooled results. Filter plots were generated to determine whether publication bias was present if there were ten or more primary studies. P<0.05 was considered to indicate statistical significance.

### Mendelian randomization analysis

### Basic concept of MR analysis

Regarding reverse causation and confounding, MR analysis is less vulnerable to bias than traditional observational methods because genetic variants are randomly distributed during gamete formation and are not correlated with environmental factors. Single-nucleotide polymorphisms (SNPs) linked to hemodialysis patient outcomes and death were identified in this study using MR analysis. Once the SNPs were identified, they were combined to evaluate the association between the risk of hemodialysis and significant factors linked to mortality during treatment. To ensure valid causal estimation, the following requirements must be met for each SNP to be used as an instrumental variable in MR analysis: (1) the genetic variation must be associated with significant hemodialysis mortality factors; (2) it cannot be affected by confounding factors; and (3) it must only affect the risk of hemodialysis through significant factors related to hemodialysis death.

### Data sources

We used publicly available summary statistics from large-scale GWAS datasets in our two-sample MR analysis. As shown in Table 1, summary statistics for trauma infection and essential nutrients were retrieved from a GWAS database (http://gwas.mrcieu.ac.uk/website). The selected SNPs must have a strong correlation with type 2 diabetes (T2D) incidence, dialysis status, age, zinc concentration, and serum phosphorus concentration for use in MR. We defined F > 10 (F = N - K - 1/K X R2/1 - R2) as indicating no weak instrumental bias to guarantee a strong correlation between the selected SNPs and outcomes and prevent the bias of weak instrumental variables. R2 (the percentage of variance in the exposure database explained by SNPs) was calculated by the formula R2 = 2 X (1 - MAF) X MAF X (β/SD); N and K are the sample sizes in the exposure database. The formula for calculation includes the secondary allele frequency (MAF), the allele effect value (β), and the standard value (SD).

**Table 1:** Detailed information on single nucleotide polymorphisms (SNPs) related to leakage factors and outcome factors.

	GWAS ID	Trait	Consortium	Sample Size	Number of SNPs
Type 2 diabetes	ebi-a-GCST006867	Type 2 diabetes	NA	655,666	5,030,727
Age	ebi-a-GCST90000050	Age at first birth	NA	542,901	9,702,772
Serum phosphorus	bbj-a-45	Phosphorus	NA	42,793	6,108,953
hemodialysis	finn-b-N14_DIALYSIS	Dialysis	NA	—	16,380,451

### MR analysis

This work employs the inverse-variance weighting (IVW) method as its main methodology. IVW is well known for its resilience in identifying causal relationships. However, it presupposes that exposure affects the outcome and requires significant genetic variation. Four additional techniques are used to improve the accuracy of MR analysis: weighted mode, simple mode, weighted median estimator (WME), and MR Egger regression. High reliability was indicated by consistent results from all five of these MR analysis techniques. A value close to 0 indicates that horizontal pleiotropy is absent. This is the meaning of the intercept term in MR Egger regression. Significant P values indicate heterogeneity. The Cochran Q test was used to measure heterogeneity. A one-method exclusion analysis is carried out to guarantee the effects' general reliability. The study employs a significance level (α) of 0.05 for the results, which are displayed as odds ratios (ORs) and 95% confidence intervals (CIs). The R (v4.1) functions 'TwoSample-MR' and 'MRPRESSO' were used in all analyses.

## Results

### Basic data from the meta-analysis

Sixteen literary works were ultimately selected for analysis. There were 3,87025 patients in the total sample, and 30 important risk factors were identified. Table 2 displays the specifics of the included studies.

**Table 2:** Literature details were included. Annotate: 1. Age; 2. BMI; 3. BSI-free days 4. Cardiothoracic ratio; 5. Charlson index; 6. Cholesterol; 7. Comorbidities; 8. Creatinine; 9. CVD; 10. Diabetic; 11. Dialysis session length; 12. Dyslipidemia; 13. Hemodialysis vintage; 14. ESI-free days 15. Hemoglobin; 16. Hypertension; 17. Infection; 18. Kidney disease; 19. Kt/V; 20. Non-use of VDRAs; 21. nPCR; 22. Peripheral vascular disease; 23. Serum albumin; 24. Serum corrected calcium; 25. Serum CRP; 26. Serum ferritin; 27. Serum Glucose; 28. Serum intact PTH; 29. Serum phosphorus; 30. Sex; 31. Smoke; 32. Stroke.

Number	First Author	Article Type	Sample<br>size	Reported<br>outcomes	Country	Publication<br>time	quality<br>score	Risk Factors
1	Klinger Mr [9]	Review	61	All-cause mortality and cardiac death	Canada, USA	2019	7	1,6,23
2	Vicentini CAA [10]	Follow up research	592	All-cause mortality and cardiac death	Brazil	2023	8	1,3,7,8,10,14,21
3	Hiyamuta H [11]	Follow up research	3505	All-cause mortality and cardiac death	Japan	2020	6	1,2,4,6,9,10,11,13,15,16,19,20,23,24,25,26,28,29,30
4	Nakaya R [12]	Follow up research	516	All-cause mortality and cardiac death	Japan	2022	7	9,17
5	Abe M [13]	Follow up research	203008	All-cause mortality and cardiac death	Japan	2021	7	2,13,19,21,23,30
6	Gil Giraldo Y [14]	Prospective observational study	100	All-cause mortality and cardiac death	Spain	2020	8	1,5,9,10,12,13,15,16,22,23,30,32
7	Yotsueda R [15]	Q-Cohort Study	3436	All-cause mortality and cardiac death	Japan	2017	8	1,9,26,16,30
8	Almeida FA [16]	Prospective obser­ vational study	334	All-cause mortality and cardiac death	Brazil	2010	6	1,8,15,16,27
9	Xiang F [17]	Follow up research	355	All-cause mortality and cardiac death	China	2017	9	1,2,6,9,10,11,15,16,23,24,30,31
10	Jung YSg [18]	Follow up research	120	All-cause mortality and cardiac death	Korea	2014	7	1,10,16,19,30
11	Xiang F [17]	Follow up research	162360	All-cause mortality and cardiac death	Japan	2020	9	1,9,10,13,18,30
12	Hall RK [19]	Follow up research	3500	All-cause mortality and cardiac death	USA	2018	7	1,5,11,15,19,30
13	Abbott KC [20]	Follow up research	6939	All-cause mortality and cardiac death	USA	2018	7	1,2,9,10
14	Fleischmann E [21]	Follow up research	1346	All-cause mortality and cardiac death	USA	1999	6	1,2,8,10,23,30
15	Wu HC [22]	Retrospective study	176	All-cause mortality and cardiac death	China	2017	8	2,10,15,16,19,23,25,30
16	Amaral S [23]	Retrospective study	677	All-cause mortality and cardiac death	USA	2006	6	1,23,30

### Meta-study results

### Results of the random-effects model analysis

Significant heterogeneity was observed (P<0.001, I^2^ = 99.5%) in the death outcomes that were reported in all 16 hemodialysis studies. Galbraith's star chart indicated that certain studies were outside the confidence interval and that the slope of the scatter plot was significant. The Baujat plot continued to show heterogeneity even after the heterogeneous portion was removed. Thus, for the meta-analysis, the random-effects model was applied. Several significant variables, including BMI, age, sex, diabetes status, CVD incidence, hemoglobin status, hypertension status, Kt/V hemodialysis vintage, serum CRP concentration, number of dialysis sessions, and CCI, were supported by multiple studies according to the results of the random-effects model meta-analysis (I^2^=0.913, P=0.181).

### Analytical results of the hierarchical random-effects model

Stratified analysis was used to further analyze the meta-analysis results because it can significantly reduce heterogeneity. The heterogeneity did not significantly differ (P=0.1078, I^2^=91.06%), suggesting that the heterogeneity increased with the combination of various factors. Several variables that were found to be significant by multiple studies were revealed by the results of the meta-analysis. The length of the dialysis session, serum CRP concentration, CCI, T2D status, serum phosphorus concentration, and serum albumin concentration were also recorded (Table 3).

**Table 3:** Meta-analysis results of a stratified random effects model.

Variable	estimate	se	zval	pval	ci.lb	ci.ub
intrcpt	0.171	0.077	2.223	0.026	0.020	0.321
BMI	-0.263	0.132	-1.990	0.047	-0.523	-0.004
Cardiothoracic ratio	0.020	0.281	0.071	0.943	-0.531	0.571
Charlson index	-0.066	0.214	-0.307	0.759	-0.485	0.354
Cholesterol	-0.363	0.185	-1.968	0.049	-0.725	-0.001
Comorbidities	0.012	0.291	0.040	0.968	-0.559	0.582
Creatinine	-0.102	0.193	-0.527	0.598	-0.480	0.277
CVD	0.234	0.142	1.644	0.100	-0.045	0.512
Diabetic	0.185	0.139	1.337	0.181	-0.086	0.457
Dialysis session length	-0.118	0.176	-0.673	0.501	-0.462	0.226
Dyslipidemia	0.235	0.608	0.386	0.699	-0.956	1.426
Hemodialysis vintage	-0.205	0.154	-1.332	0.183	-0.506	0.097
Hemoglobin	-0.258	0.136	-1.893	0.058	-0.524	0.009
Hypertension	-0.107	0.149	-0.719	0.472	-0.398	0.184
Infection	-1.444	0.428	-3.372	0.001	-2.283	-0.604
Kidney disease	-0.002	0.259	-0.006	0.995	-0.510	0.507
Kt/V	-0.305	0.159	-1.926	0.054	-0.616	0.005
Non-use of VDRAs	-0.031	0.309	-0.100	0.921	-0.637	0.576
nPCR	-0.570	0.275	-2.069	0.039	-1.109	-0.030
Peripheral vascular disease	-0.527	0.718	-0.735	0.463	-1.934	0.880
Serum albumin	-0.593	0.133	-4.454	0.000	-0.854	-0.332
Serum corrected calcium	-0.141	0.219	-0.644	0.519	-0.570	0.288
Serum CRP	-0.017	0.205	-0.081	0.935	-0.418	0.385
Serum ferritin	-0.171	0.273	-0.625	0.532	-0.706	0.365
Serum Glucose	1.183	0.442	2.677	0.007	0.317	2.049
Serum intact PTH	-0.171	0.273	-0.624	0.532	-0.706	0.365
Serum phosphorus	-0.031	0.280	-0.110	0.912	-0.579	0.517
Sex	-0.052	0.125	-0.414	0.679	-0.296	0.193
Smoke	-0.221	0.352	-0.628	0.530	-0.910	0.469
Stroke	0.523	0.740	0.706	0.480	-0.927	1.972

### Sensitivity analysis and deviation testing

The horizontal pleiotropy test results (T=0.39, df=116, P=0.6953) indicated that there was no horizontal pleiotropy. the results of the sensitivity analysis (P=0.1186, I^2^=99.53%) demonstrated the general stability of the data.

### Mendelian randomization analysis

According to the GWAS data, no SNPs with significant differences in meta-analysis scores, including session length, serum CRP concentration, or CCI, were detected. Thus, the analysis focused solely on the associations of T2D incidence, serum phosphorus concentration, and serum albumin concentration with hemodialysis.

### Selection results of IVs

Nineteen SNPs without linkage imbalance were identified from the exposure datasets for serum albumin, age, T2D status, and serum phosphorus (rs13234269, rs1758632, rs6494307, rs2058913, rs10752613, rs13420733, rs4443016, rs62261746, rs13319205, rs2530597, rs1464534, rs1590949, rs7958796, rs7359501, rs10445366, rs5763436, rs733323, rs2389858, and rs2001945). These SNPs were selected as instrumental variables to assess their relationships with hemodialysis. The F-statistics for these SNPs ranged from 63.14 to 148.48 (Serum albumin), 19.39 to 261.55 (age), 26.13 to 92.40 (T2D), and 51.93 to 126.16 (Serum phosphorus).

### Age, serum albumin concentration, type 2 diabetes incidence, serum phosphorus concentration, and hemodialysis Mendelian randomization results

**Figure 2:**
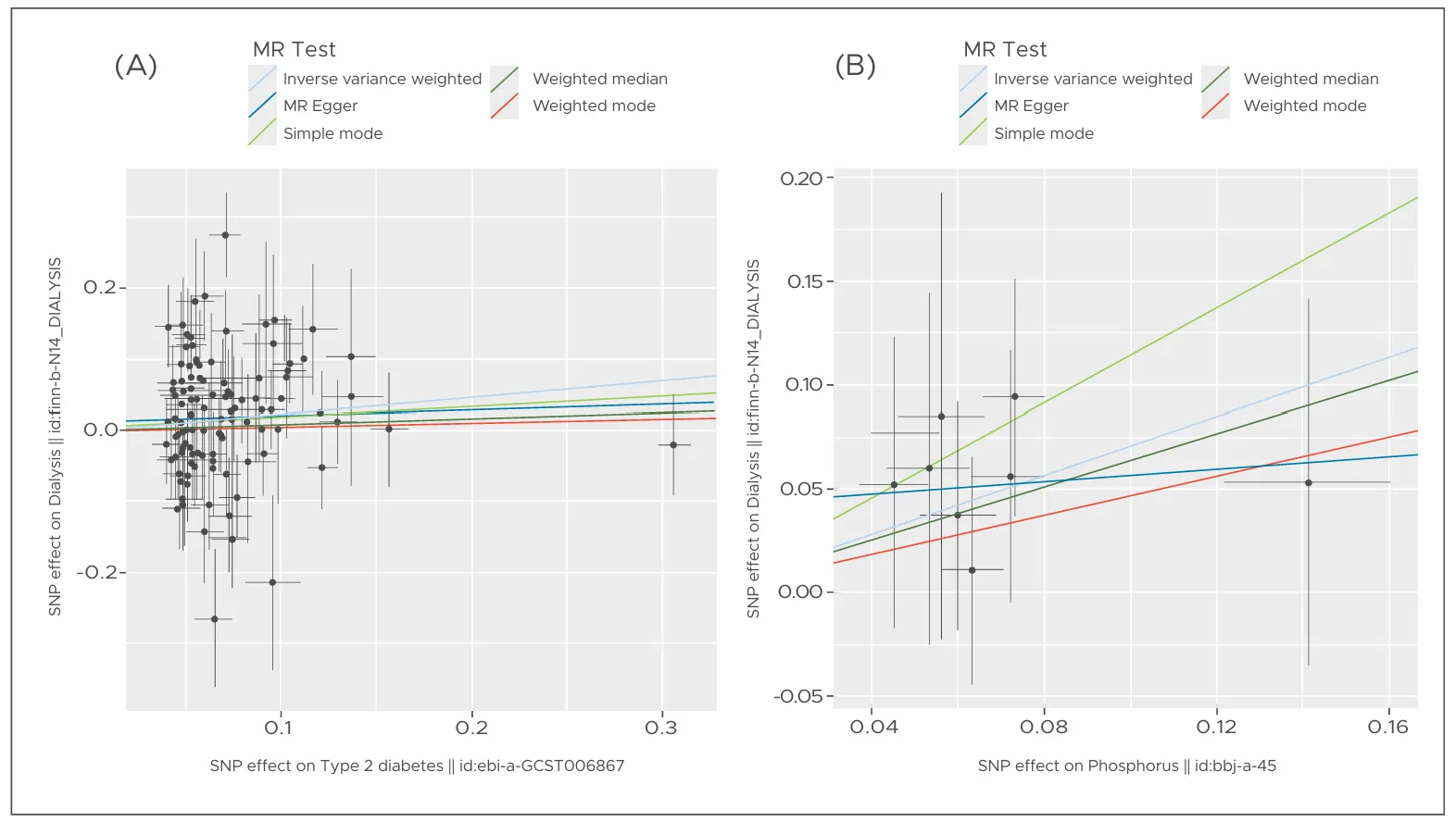
Shows scatter plots of the causal effect (A: Type 2 diabetes and hemodialysis; and B: Serum phosphorus and hemodialysis).

The IVW results indicated a positive causal relationship between T2D and hemodialysis at the genetic level (OR=1.2572, 95% CI = 1.0375-1.5235; P=0.0195). There was also a positive correlation between the serum phosphorus concentration and hemodialysis status at the genetic level (OR=2.0269, 95% CI = 1.0614-3.8708; P=0.0323). However, age (OR=1.1112, 95% CI=0.8395-1.4707; P=0.4612) and serum albumin concentration (OR=1.2933, 95% CI=0.8931-1.8729; P=0.1733) did not show a positive causal relationship at the gene level with hemodialysis. The specific results are presented in Table 4. The IVW results were validated through MR Egger, WME, weighted mode, and simple mode analyses. The scatter plot confirmed that the regression lines for age predicted by genetics, T2D status, serum phosphorus concentration, and serum albumin concentration were largely consistent with the risk of hemodialysis, indicating the reliability of the MR analysis results, as shown in Figure 2.

**Table 4:** OR estimates for Inverse Variance Weighted (IVW), Mendelian Randomization (MR) Egger regression, Weighted Median Estimator (WME), Weighted Mode, Simple Mode, and 95% confidence interval (CI).

Exposure	Outcome	Number<br>of SNPs	Method	SE	P	OR	95%CI	
Type 2 diabetes	hemodialysis	114	MR Egger	1.0682	0.7292	1.0843	0.6865	1.7127
		114	Weighted median	0.3999	0.5571	1.0836	0.8288	1.4169
		114	Inverse variance weighted	0.3301	0.0195	1.2572	1.0375	1.5235
		114	Simple mode	0.558	0.6202	1.1611	0.6441	2.0931
		114	Weighted mode	0.4849	0.7558	1.0512	0.7679	1.4392
Serum phosphorus	hemodialysis	8	MR Egger	1.0682	0.8919	1.1636	0.1434	9.4412
		8	Weighted median	0.3999	0.1096	1.8962	0.8659	4.1521
		8	Inverse variance weighted	0.3301	0.0323	2.0269	1.0614	3.8708
		8	Simple mode	0.558	0.0795	3.1396	1.0518	9.372
		8	Weighted mode	0.4849	0.3649	1.5996	0.6184	4.1373
Serum albumin	hemodialysis	224	MR Egger	0.3569	0.6586	1.1711	0.5818	2.3574
		224	Weighted median	0.3116	0.2808	1.3995	0.7598	2.5778
		224	Inverse variance weighted	0.1889	0.1733	1.2933	0.8931	1.8729
		224	Simple mode	0.6764	0.6690	1.3358	0.3548	5.0293
		224	Weighted mode	0.3453	0.5370	1.2381	0.6292	2.4361
Age	hemodialysis	55	MR Egger	0.5909	0.7059	1.2512	0.3930	3.9837
		55	Weighted median	0.1928	0.3825	1.1834	0.8110	1.7268
		55	Inverse variance weighted	0.1430	0.4612	1.1112	0.8395	1.4707
		55	Simple mode	0.5127	0.3833	0.6372	0.2333	1.7406
		55	Weighted mode	0.4279	0.3278	1.5257	0.6596	3.5292

### Horizontal pleiotropy analysis and heterogeneity test

**Figure 3:**
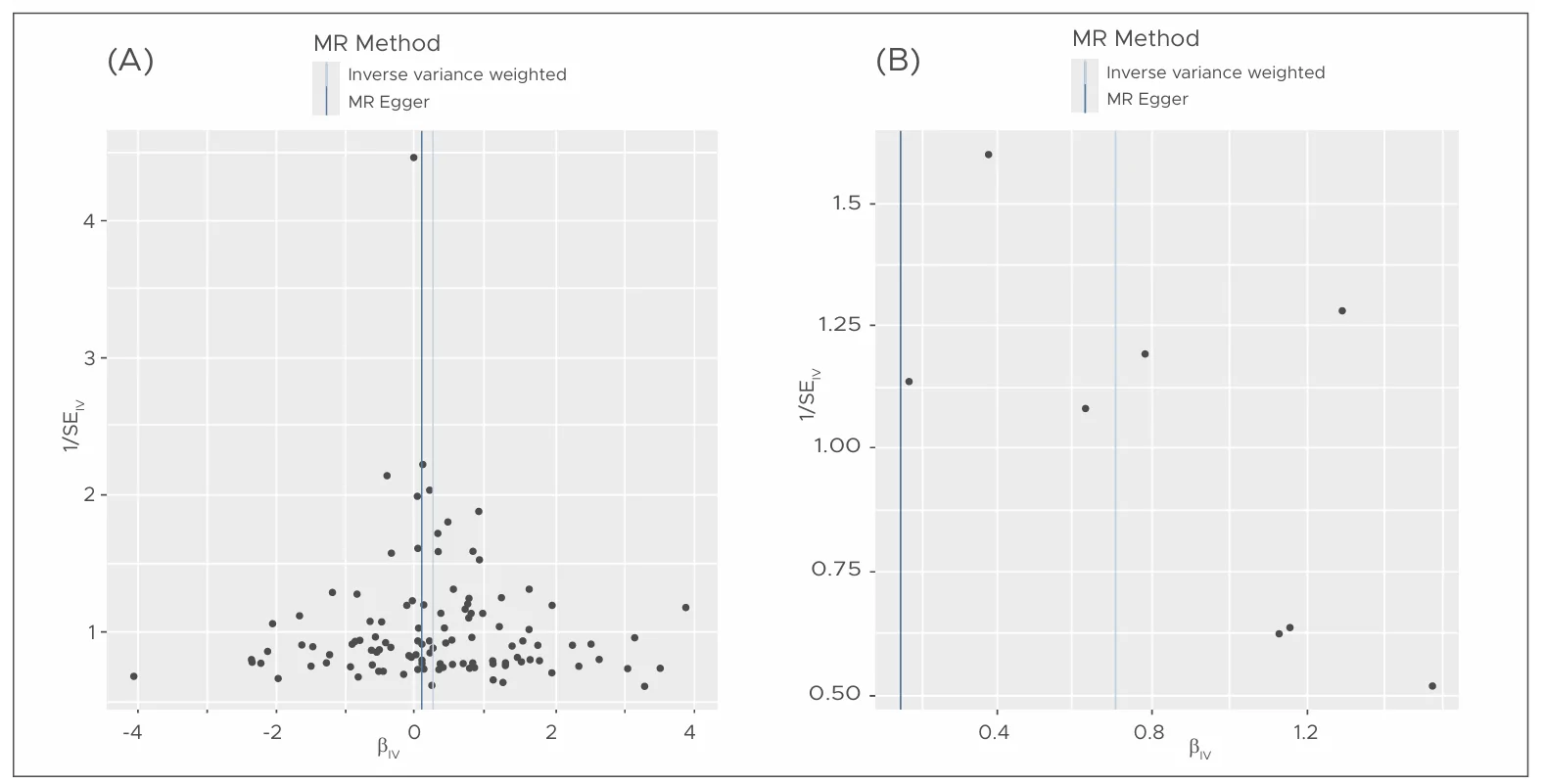
Funnel plot of causal effect relationship (A: Type 2 diabetes and hemodialysis; and B: Serum phosphorus and hemodialysis).

According to the results of the MR Egger intercept analysis, no potential pleiotropy was found for age (P = 0.8366), serum albumin concentration (P=0.7432), T2D status (P=0.6045), or serum phosphorus concentration (P=0.4857). This finding implies that other than exposure, instrumental variables had no discernible impact on the outcomes. Potential heterogeneity in the serum phosphorus concentration was indicated by Cochran's Q heterogeneity test (IVW P=0.0035, Mr Egger P=0.0032). Age (IVW P=0.1047, MR Egger P=0.0895) and serum albumin concentrations in patients with T2D (IVW P=0.9803, MR Egger P=0.9739) did not significantly affect the outcome. Table 5 illustrates that no discernible heterogeneity was found. The leave-one-out method confirmed that the overall causality estimation was unaffected by any individual SNP A symmetrical funnel plot that illustrates the distribution of causal effects implies that there are no underlying factors influencing the outcomes. The outcome is shown in Figure 3.

**Table 5:** Sensitivity analysis of various MRI studies.

Exposure	Outcome	Cochran Q		MR Egger		MR-PRESSO	
		MR Egger	IVW	intercept	P	Outlier	P
Type 2 diabetes	hemodialysis	P=0.9739	P=0.9803	0.0418	0.6045	0.017	0.486
Serum phosphorus	hemodialysis	P=0.0032	P=0.0035	0.0117	0.4857	0.077	0.605
Age	hemodialysis	P=0.0895	P=0.1047	-0.0084	0.8366	0.040	0.836
Serum albumin	hemodialysis	P=0.1837	P=0.1950	0.0028	0.7432	0.009	0.743

## Discussion

Thirty-two risk factors were linked to mortality in hemodialysis patients based on our analysis of sixteen studies. The serum CRP concentration, CCI, dialysis status, T2D status, serum phosphorus concentration, and heterogeneity were among the significant factors we identified after performing a meta-analysis using both random- and stratified-effects models. Reduced serum albumin concentrations are important risk factors linked to hemodialysis patient deaths. A genetic causal relationship was found between the serum phosphorus concentration and the risk of hemodialysis according to the results of MR analysis, with T2D, age, the serum albumin concentration, and the serum phosphorus concentration as critical factors and hemodialysis as the result. Consequently, this study used MR and meta-analysis to demonstrate that T2D and serum phosphorus levels are significant risk factors for hemodialysis occurrence and are positively correlated with death in hemodialysis patients.

Our study aimed to identify mortality risk factors in hemodialysis patients using a systematic review and meta-analysis, and to investigate the causal relationships between serum phosphorus, albumin, and CRP and hemodialysis risk using Mendelian randomization analysis. Our results identified serum phosphorus, albumin, and CRP as mortality risk factors in hemodialysis patients, consistent with previous studies [24][25]. The Mendelian randomization analysis revealed a positive causal relationship between serum phosphorus and hemodialysis risk, suggesting that high serum phosphorus levels may increase the risk of mortality in hemodialysis patients. Our findings are consistent with a recent study by Yan et al. [24], which found that genetically predicted reduced glomerular filtration rate was associated with higher odds of appendicular lean mass and grip strength, suggesting a potential causal relationship between renal function and sarcopenia [24]. Similarly, our study found that serum phosphorus, a marker of renal function, was positively associated with hemodialysis risk. Another study by Zhou et al. [25] found that increased serum iron, ferritin, and transferrin saturation were associated with elevated immunoglobulin A nephropathy risk, suggesting a potential causal effect of systemic iron status on kidney disease [25]. Our study did not investigate the relationship between iron status and hemodialysis risk. A genome-wide association study by Siew et al. [26] identified two novel loci associated with acute kidney injury, including a locus near the FTO gene that was attenuated after adjustment for BMI and diabetes. Our study did not investigate the relationship between genetic variants and hemodialysis risk. A Mendelian randomization study by Hou et al. [27] found that N-acetylleucine and glycine to alanine ratio were associated with lower and higher risk of chronic kidney disease, respectively. Our study did not investigate the relationship between metabolites and hemodialysis risk.

According to the consensus statement issued by the Swiss Society of Diabetes and Nephrology, the main cause of end-stage renal disease in people with T2D is their heightened risk of developing diabetic nephropathy. Improving early screening for diabetic nephropathy and increasing clinicians' knowledge of the condition is crucial. Early clinical intervention can lessen patients' financial burden while also improving clinical outcomes [28]. Diabetes increases the risk of frailty in end-stage renal disease patients undergoing hemodialysis treatment according to the findings of a systematic review and meta-analysis. Additionally, patients with frailty had a greater risk of death than patients without frailty (OR=2.02, 95% CI = 1.65-2.48) [29]. In a study examining risk factors associated with death in patients undergoing hemodialysis treatment or peritoneal dialysis for end-stage renal disease and T2D, high cholesterol levels at the start of peritoneal dialysis treatment were found to be independent risk factors for death. A lower BMI and higher HbA1c level were risk factors linked to mortality during hemodialysis treatment. Nursing care for patients with end-stage renal disease or T2D who are undergoing hemodialysis treatment should be customized based on the patient's nutritional status. To reduce and prevent the risk of death and disease progression in these patients, targeted nursing programs should be created. Simultaneously, it is crucial to track variations in HbA1c levels as the nursing process progresses. Patients should be closely monitored, and the nursing and clinical treatment plan should be modified if there is a persistent or abrupt increase in HbA1c [30].

Dietary sources or endogenous production are the sources of serum phosphorus. Fibroblast growth factor-23 (FGF-23) and parathyroid hormone (PTH) are both stimulated by phosphorus. Both phosphaturic hormones, FGF-23 and PTH, promote the absorption of phosphate from the blood via NaPi-2a on the apical membrane of renal tubules. This mode of absorption promotes phosphorus excretion and limits the reabsorption of phosphate in the urine. Hyperphosphatemia and elevated FGF-23 expression are frequently observed in patients with chronic kidney disease. In cardiomyocytes, elevated expression levels of FGF-23 stimulate the transcription of genes linked to pathological hypertrophy, resulting in left ventricular hypertrophy. Hyperphosphatemia and Klotho deficiency are concomitant with reduced endothelial cell function, increased calcification of the arterial wall and valvular disease. Hyperphosphatemia and elevated FGF-23 expression are the main risk factors for cardiovascular disease development in individuals with chronic kidney disease [31][32]. The main cause of death for patients with chronic kidney disease undergoing hemodialysis treatment is concurrent cardiovascular disease. Intensive hemodialysis management has been shown to lower patient mortality by lowering the risk of adverse cardiac events [33].

## Conclusion

According to our research, T2D and serum phosphorus are significant independent risk factors for mortality in hemodialysis patients as well as hemodialysis patients with end-stage renal disease. Patients suffering from hyperphosphatemia and diabetic nephropathy require close observation and monitoring throughout their clinical and nursing care.

## Dodatak

### Author contributions

All authors have made substantial contributions to the conception and design of the study. Xiaofen Ma has been involved in data analysis, data interpretation, drafting the manuscript, and revising it critically. Huan Ye, and Xiaofen Ma have been involved in designing the study and revising the manuscript critically. Huan Ye gave the final approval for the version to be published.

### Acknowledgements

We thank the researchers for sharing their data on the GWAS data.

### Ethics statement

Each cohort included in this study received its respective institutional research ethics board's approval to enroll patients and all participants provided written informed consent. All information used for this study is publicly available as deidentified GWAS summary statistics.

### Conflict of interest statement

All the authors declare that they have no conflict of interest in this work.
